# Promoting professional identity, motivation, and persistence: Benefits of an informal mentoring program for female undergraduate students

**DOI:** 10.1371/journal.pone.0187531

**Published:** 2017-11-01

**Authors:** Paul R. Hernandez, Brittany Bloodhart, Rebecca T. Barnes, Amanda S. Adams, Sandra M. Clinton, Ilana Pollack, Elaine Godfrey, Melissa Burt, Emily V. Fischer

**Affiliations:** 1 Department of Learning Sciences and Human Development, West Virginia University, Morgantown, West Virginia, United States of America; 2 Department of Atmospheric Sciences, Colorado State University, Fort Collins, Colorado, United States of America; 3 Environmental Program, Colorado College, Colorado Springs, Colorado, United States of America; 4 Department of Geography and Earth Sciences, University of North Carolina at Charlotte, Charlotte, North Carolina, United States of America; International Nutrition Inc, UNITED STATES

## Abstract

Women are underrepresented in a number of science, technology, engineering, and mathematics (STEM) disciplines. Limited diversity in the development of the STEM workforce has negative implications for scientific innovation, creativity, and social relevance. The current study reports the first-year results of the PROmoting Geoscience Research, Education, and SuccesS (PROGRESS) program, a novel theory-driven informal mentoring program aimed at supporting first- and second-year female STEM majors. Using a prospective, longitudinal, multi-site (i.e., 7 universities in Colorado/Wyoming Front Range & Carolinas), propensity score matched design, we compare mentoring and persistence outcomes for women in and out of PROGRESS (*N* = 116). Women in PROGRESS attended an off-site weekend workshop and gained access to a network of volunteer female scientific mentors from on- and off-campus (i.e., university faculty, graduate students, and outside scientific professionals). The results indicate that women in PROGRESS had larger networks of developmental mentoring relationships and were more likely to be mentored by faculty members and peers than matched controls. Mentoring support from a faculty member benefited early-undergraduate women by strengthening their scientific identity and their interest in earth and environmental science career pathways. Further, support from a faculty mentor had a positive indirect impact on women’s scientific persistence intentions, through strengthened scientific identity development. These results imply that first- and second- year undergraduate women’s mentoring support networks can be enhanced through provision of protégé training and access to more senior women in the sciences willing to provide mentoring support.

## Introduction

In the United States, women continue to be underrepresented in many science, technology, engineering, and mathematics (STEM) disciplines [1], including the earth and environmental sciences [2]. Limited workforce diversity has negative implications for scientific innovation, creativity, and social relevance [3]. The underrepresentation of women in is particularly severe in earth and environmental science-related majors [1, 4]. For a time, the percentage of female students earning baccalaureate degrees in earth and environmental science related-majors was on the rise, but this number peaked in 2004 at 38.7% and has since dropped to 36% [5]. It is critical to better understand the underlying causes of a lack of diversity in STEM, broadly, as well as issues specific to particularly un-diverse fields. Strategies that promotes greater representation from all genders are needed. The current study reports on the impact of a novel informal mentoring program aimed at supporting first- and second-year undergraduate female STEM majors’ motivation and persistence by fostering greater scientific identity.

### Mentoring undergraduates

A variety of strategies have been proposed to support students’ persistence in scientific career pathways, and mentoring support is typically a critical element of comprehensive intervention strategies [6–11]. We define mentoring as a developmental relationship between a more experienced person (i.e., the mentor) and a less experienced person (i.e., the protégé), where the mentor’s aim is to support the protégé’s professional development and socialization into the profession [12–14]. Mentoring relationships can be formed through formal programs, but are most often informal in research with undergraduates [13, 15]. At the undergraduate level, mentorship typically involves one or more active support functions: psycho-social-emotional support (i.e., counseling, guidance, and encouragement), instrumental support (i.e. skill development through assistance on challenging tasks and opportunities for advancement), or coauthoring experiences (i.e. collaborative presentations or publications of research) [16–18]. Mentorship can also involve passive support when a protégé perceives the mentor as an outstanding career role model [14, 19]. In contrast to active supports, *role modeling* support involves serving as an inspiration and example of success, as well as, being a guide for the norms, behaviors, and values that are needed to succeed [19]. Meta-analytic and primary evidence indicate that mentoring support is the processes through which mentoring affects the development and success of the protégé [16, 19, 20].

#### Direct effects of mentoring on beneficial outcomes

Mentoring researchers and theorists have predicted that mentoring support should directly and positively improve protégé’s academic success, scholarly productivity, health, having a positive attitudes toward the field, professional identity development, or motivation to learn), and career outcomes or intentions [12, 14, 21]. However, a recent large-scale meta-analysis comparing outcomes across 166 studies found only partial support for the direct positive benefits of mentoring support [21]. In general, the meta-analysis found small positive benefits for students with a mentor compared to those without a mentor (e.g., mentored students had slightly higher levels of academic success [$\bar{r}$ = .19], motivation [$\bar{r}$ = .14], and lower levels of withdrawal or dropout [$\bar{r}$ = -.11]) [21]. There are, however, at least two reasons why researchers might expect to find stronger positive effects of mentoring for women in STEM.

First, advances in mentoring theory and empirical evidence indicate that failure to capture the nuanced conditional processes underlying mentoring effects may lead to underestimation of benefits [7, 12]. Second, despite a growing body of evidence on the potential benefits of mentoring for women and underrepresented racial minorities in STEM disciplines, the unique impact of mentoring has been difficult to ascertain. Much of the literature has focused on the efficacy of multicomponent interventions that simultaneously implement several support strategies (e.g., mentoring and research experiences), and disentangling the unique impact of mentoring is often untenable [9, 11, 22]. In addition, empirical studies that have isolated the impact of mentoring may have underestimated the benefits on student outcomes due to: a) measurement tools that were not theoretically-based or rigorously validated; b) study designs that failed to control for confounding variables; and/or c) focusing on a highly selective group of college juniors or seniors in the context of undergraduate research experiences [10, 12, 21, 23–29].

#### Mentoring college women

Mentoring theory and empirical evidence indicate that having a broad network with multiple developmental mentoring relationships can be particularly important and helpful for women pursuing scientific and professional careers [30–35]. For women in male-dominated fields, research indicates broader networks of mentors are associated with experiencing the full benefits of mentoring [36]. For example, a study of predominantly upper-division female science majors examined the prevalence and influence of numerous developmental relationships (i.e., mentors, role models, sponsors), as well as their influence on choosing a science major [30]. The study indicated that female science majors had, on average, six developmental relationships and revealed that multiple types of developmental relationships (i.e., mentors and role models) had a positive influence on choosing a science major. However, research has also indicated that compared to upper-division college women, lower-division college women have small networks of developmental mentoring relationships and they tended to only seek out a single mentor [34]. Therefore, we hypothesize that the positive benefits of mentoring may only be realized when lower-division college women in STEM disciplines have access to broader networks.

Beyond having multiple mentors, research indicates that receiving support from same-gender mentors and role models is particularly important for women in STEM [19, 23]. Theory and evidence indicate that female undergraduates with a female faculty mentor report receiving higher levels of mentoring support compared to female undergraduates with a male faculty mentor [17, 23]. In addition, female mentors that are members of the scientific community can serve as role models, supporting future career aspirations for female undergraduate STEM majors [19, 37]. Research indicates that same-gender and counter-stereotypic STEM career role models can reduce women’s perceptions of barriers to fitting into STEM careers [38–42].

### The role of identity

#### Mentoring as an intervention to promote scientific identity, motivation, and persistence

Recent advances in STEM career development theory suggests that the most successful intervention strategies promote professional identity development as a means of enhancing motivation and persistence in STEM [7, 20, 43]. Developing a strong scientific identity involves students coming to see themselves as a scientist, feeling a sense of belonging in the scientific community, and receiving recognition from important scientific mentors and role models [44–46].

STEM career development theory suggests that professional identity development enhances student motivation to learn and also their long-term persistence in STEM [7]. Empirical research with college STEM majors has shown that scientific identity development enhances some types of motivation, such as achievement goals and competence beliefs [47, 48], but the links to other relevant motivational processes is not well established. One highly relevant theoretical model for STEM career development frames motivation in terms of four phases of interest development, where interest is defined as a psychological process of engaging and re-engaging with particular content (e.g., STEM objects, ideas, or events) over time [49]. The model posits that individuals move from fleeting forms of interest that are initially sparked by an external event or cue (i.e., situational interest [Phase 1]) to deeper, more personal, and enduring levels of interest (i.e., maintained interest [Phase 2], emerging individual interest [Phase 3], and ultimately developed individual interest [Phase 4]; [49]. Although research indicates that curricular interventions can enhance student interest in STEM disciplines [50, 51], the empirical link from identity development to interest development is yet to be established. In addition to motivation, research indicates that scientific identity development supports persistence in STEM career pathways (e.g., intentions to pursue a scientific career, graduate school matriculation, post-graduate STEM career attainment) [43, 52–54]. Therefore, we hypothesized that mentoring will operate through scientific identity to promote deeper levels of interest in earth and environmental sciences (i.e., motivation) and scientific persistence intentions.

### Current study

The purpose of the current study is to examine the unique benefits a novel informal mentoring program aimed at supporting first- and second-year female STEM majors’ scientific identity development, deep interest in earth and environmental sciences, and pursuit of scientific career pathways. We report initial findings from the PROmoting Geoscience Research, Education, and SuccesS (PROGRESS) program, which forms the basis for an ongoing, prospective, longitudinal, multisite propensity score matched study of female STEM students’ academic journeys. Data reported here were collected from students at one-of-seven four-year universities in the Colorado/Wyoming Front Range or the Carolinas. Participant recruitment and data collection began in the fall semester of 2015 and follow-up survey data collection was completed in the spring of 2016. In fall semester of 2015, students completed a brief matching survey of background, demographics, psychological characteristics related to STEM (i.e., beliefs, perceptions, values), and scientific career aspirations, and were recruited into the PROGRESS program. PROGRESS members participated in a weekend workshop (Fall 2015), were given access to a secure online peer support community, and were connected with female scientific mentors in their community (e.g., faculty, postdoctoral researchers, graduate students, or professional scientists).

In addition to PROGRESS participants, we recruited a Propensity Score Matched (PSM) sample of first- and second-year female STEM majors that did not participate in the PROGRESS program (complete details provided in the Procedures section below). The PSM matched group serves as a quasi-experimental “inactive” or treatment as usual control group [55, 56]. In the present context, treatment as usual means that controls did not participate in the weekend workshop and only received mentoring support as typically provided in their college environments. Comparisons of treatment and inactive control groups allow for the assessment of an overall impact [55]; however, the use of a treatment as usual control group makes it difficult to draw conclusions about particular ingredients of PROGRESS [56].

**Fig 1:**
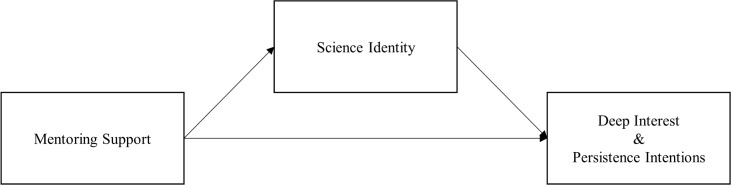
Conceptual mediation model linking mentoring support to motivation and persistence through professional identity development.

Our longitudinal matched design allows us to address two central research questions about the benefits of mentoring lower-division female STEM majors: 1) Does participation in PROGRESS enhance women’s network of scientific mentors (i.e., faculty, non-faculty scientific professionals, graduate students, & peers)? 2) To what degree does mentoring support women’s scientific identity development, deep-level interest in earth and environmental science education and careers (i.e., motivation), and scientific persistence intentions? Further, we explored the degree to which positive benefits of mentorship on deep interest and persistence intentions were mediated through scientific identity (see conceptual model presented in Fig 1).

## Materials and methods

To participate in the longitudinal study, students read and agreed to an IRB approved informed consent form. Colorado State University Institutional Review Board approved this study. The approved protocol number is 14-4829H.

### Participants

The total sample in the overall study consisted of 240 first- and second-year college female STEM majors recruited from seven universities in the Colorado/Wyoming Front Range and the Carolinas. For this particular part of the study, we focused on an analytic sample of 116 propensity score-matched first- and second-year students majoring in a variety of STEM disciplines (*n*_*PROGRESS*_ = 58; *n*_*Control*_ = 58), see S1 Table.

### Procedure

Students were recruited via email (i.e., email addresses obtained in cooperation with university registrar offices; department listserves, or individual science faculty forwarding email solicitation to students in their classes), in-person recruitment announcements in introductory STEM courses (e.g., Physics I), and flyers advertising the study posted across campus. To participate in the longitudinal study, students completed a brief online matching survey (including consent form), and received a nominal gift ($5 Starbucks eGiftcard) for their participation. The brief matching survey measured student academic interests and achievements, demographic characteristics, family support and resources, interest in having a mentor, interest in participating in a longitudinal study, and a variety of psychological and motivational factors associated with persistence in STEM (e.g., science self-efficacy).

Based on the brief matching survey, all students who met the inclusion criteria (i.e., female, STEM major, first or second year of college) were emailed a personalized invitation to participate in one of two PROGRESS weekend workshops (i.e., one in Colorado and the other in South Carolina). Eighty-five students (~35% of those invited) accepted the invitation and participated in a PROGRESS weekend workshop (*n*_*Front Range*_ = 54, *n*_*Carolinas*_ = 31).

Weekend workshops were modeled after successful workshops developed for the Earth Science Women’s Network (ESWN; [31, 57]. The goals of the workshops were to 1) train participants to assess their network of developmental mentoring relationships (mentoring support mapping exercise), develop mentoring initiation and relationship management skills (e.g., communication with mentors or prospective mentors and developing adequate expectations for support), and provide access to a network of on- and off-campus mentors to broaden their support network, 2) familiarize participants with a newly developed secure website to continue their contact with peers in PROGRESS and scientific mentors, 3) introduce participants to female role models with diverse careers in the earth and environmental sciences (i.e., panel discussions with diverse scientists inside and outside of academia), and 4) introduce participants to challenges women in STEM disciplines may face during their undergraduate education, as well as ways to cope with potential gender bias (e.g., WAGES game [58]; workshop materials available upon request). After completing the workshop, PROGRESS participants were connected with mentors from their home region from the list provided on the PROGRESS website (http://geosciencewomen.org/). Participants and home region mentors were encouraged to meet semi-regularly with the purpose of maintaining support.

PROGRESS mentors were identified in each geographic region by project researchers who are academics in earth and environmental science fields, and asked if they would be willing to participate as mentors in the PROGRESS program. A total of 108 female scientists in a variety of careers paths (i.e., faculty members/scientists inside the university [21%], graduate students and postdocs [46%], and scientific professionals outside the university [33%]) have volunteered to participate as PROGRESS mentors. A two-hour mentor training workshop was held in the Front Range region, with a digital copy of the training emailed to those not on location. The training was administered by academic faculty with a background in mentoring and academic administration, and consisted of mentoring best practices, expectations for mentoring PROGRESS participants, mentoring ethics, and a discussion about mentoring experiences, questions, and concerns.

In the spring semester of 2016 (six months after the brief matching survey), PROGRESS and matched control participants were invited to complete an online follow-up survey concerning their educational status and achievements, educational and career aspirations, mentoring experiences, and their status on a number of relevant psychological constructs (e.g., science identity).

### Propensity score matching (PSM)

Because true randomization into the experimental condition was infeasible in this study, we constructed a matched sample of female STEM students (i.e., a quasi-experimental “inactive” or “treatment as usual” control group). However, in the absence of randomization it is understood that selection bias can occur when attributes related to self-assignment into treatment groups are also related with the outcomes of interest [59]. To control for selection bias, we constructed a matched sample of female STEM students who participated in the PROGRESS intervention and a similar sample of female STEM students who did not participate. Propensity score matching describes a family of statistical techniques designed to control for observed or measured variables that cause both the receipt of treatment and the outcome(s) of interest (i.e., confounding variables or common causes; [60]). Estimates of the treatment effect have been shown to be unbiased when the common causes of treatment and outcome (i.e., confounding variables) are included in the PSM analysis [61]. That is, PSM allows for unbiased estimates of a treatment effect when all relevant confounding variables are included in the matching process. However, the PSM estimates of treatment effects may be biased when confounding variables are unobserved or unmeasured [61].

Informed by the limitations of the PSM approach, the current study followed recommendations to collect a large amount of information on known and theoretically hypothesized causes of mentoring and persistence in STEM (e.g., demographic characteristics, family characteristics, academic preparation, desire to have a mentor, motivation, and psychological factors, etc.; see full list of covariates used in matching in S2 Table) [60]. Matched pairs were created using PSM to calculate the probability of participating in PROGRESS based on the 57 covariates (i.e., known and theoretically hypothesized causes of mentoring and persistence) measured in the brief online matching survey. The analysis revealed a 97% reduction in selection bias on the covariates used in matching, see supplemental materials and S2 Table for complete PSM analytic details.

### Measures

#### Mentoring

Participants read the following definition of mentoring: “A mentor is someone who provides guidance, assistance, and encouragement on professional and academic issues. A mentor is more than an academic advisor and is someone you turn to for guidance and assistance beyond selecting classes or meeting academic requirements.” With that definition in mind, they were asked if there was a faculty member, a graduate student, a peer, or a scientific professional outside the university that they considered a mentor.

Mentoring support from the four sources was aggregated into a single variable indicating the size of the mentoring support network: no mentor, one mentor, or multiple mentors.

#### Science identity

Science identity was measured with a three item short form of the science identity scale [43, 62]. Specifically, participants rated their agreement with each of the following statements: “In general, being a scientist is an important part of my self-image,” “I have a strong sense of belonging to the community of scientists,” and “I have come to think of myself as a ‘scientist’” on a seven-point Likert scale from strongly disagree to strongly agree. Scale scores were derived by taking the average of the three items, with higher scores indicating higher science identity. Consistent with prior studies using this scale, scores exhibited high internal consistency reliability (Cronbach’s α [63] = .86, 95% *CI* [.81, .90]).

#### Deep interest in earth and environmental sciences (Motivation)

Student motivation, operationalized as deep interest in earth and environmental sciences, was our first outcome variable. Students’ deep interest was assessed with a two-item measure adapted from prior literature on scientific interest development [51]. Participants rated their level of interest to each of the following statements: “How interested are you in taking courses in Earth Systems or Environmental Sciences?” and “How interested are you in pursuing an Earth Systems or Environmental Sciences career?” on a seven-point Likert-type scale from not at all interested to very interested. Scale scores were derived by taking the average of the two items, with higher scores indicating higher interest. Consistent with prior studies, scores exhibited high internal consistency reliability (Cronbach’s α = .92, 95% *CI* [.88, .94]).

#### Intention to pursue a scientific research career

Student’s scientific persistence intentions was our second outcome variable. We measured scientific persistence intentions with a two-item measure adapted from prior literature on scientific persistence [64]. Students rated the strength of their intentions in response to two questions: “What is the likelihood of you obtaining a science-related degree” and “To what extent do you plan to pursue a science-related research career?” on a seven point Likert-type scale from definitely will not to definitely will.

#### Analysis details

All analyses were conducted in SPSS software version 23. Prior to assessing research questions, we conducted exploratory data analysis to identify potential outliers and assess the tenability assumptions for regression analysis, see S3 Table. Outlier analysis using leverage values, standardized deleted residuals, and Cook’s D values [65–67], revealed that no cases were severe outliers. Residual diagnostics revealed that the distributions continuous outcomes were normal distributed and homoscedastic (i.e., Q-Q plots appeared normal; all Kolmogorov-Smirnov tests [68, 69] were non-significant, *p’s* > .05).

## Results

### PROGRESS impact on developmental mentoring networks

We assessed the degree to which PROGRESS enhanced student’s network of developmental mentoring relationships in two complementary sets of analyses. First, we examined the proportion of students who reported receiving mentoring support from one or more sources (i.e., faculty, graduate students, peers, or scientific professionals outside of the university). Consistent with our expectations, control group students were more likely to report having a single mentor compared to having multiple mentors, while PROGRESS students were more likely to reporting having multiple mentors compared to having a single mentor, see Table 1.

**Table 1:** Summary of mentor support descriptive statistics as a function of PROGRESS status (*N* = 116).

		Matched Control	PROGRESS
Variables		*%*	*%*
Number of science-related mentors			
	None	14	10
	One	48	29
	Multiple	38	60
Sources of mentor support			
	Faculty	24	48
	Graduate Students	16	17
	Peers	64	78
	Scientific Professional off Campus	29	35

Notes: *N* = total sample size.

A chi-square test of independence [70, 71], comparing the size of mentoring networks (no mentor, one mentor, or multiple mentors) across PROGRESS and control groups revealed that the size of mentoring networks was significantly different for PROGRESS and control groups, χ^2^(*df* = 2, *N* = 116) = 5.94, *p* = .05, *ϕ* = .23.

To fully describe the pattern of differences, we compared the odds of having one-mentor vs. no mentor and odds of having multiple mentors vs. one-mentor in the PROGRESS and control groups using hierarchical logistic regression analysis (i.e., step-1 of the hierarchical model included binary-coded indicators (0 or 1) of college campus to control for the nesting of students within schools) [72, 73]. The analyses revealed no significant difference in the odds of having one- vs. no-mentors for PROGRESS and matched control groups (*χ*^*2*^(*df* = 1) = 0.36, *p* = .54, *B* = 0.38, Odds-ratio [*O*.*R*.] = 1.46, 95% *CI* [0.43, 4.88]); however, the odds of having multiple mentors vs. one-mentor were over three-times higher in the PROGRESS group compared to the control group (*χ*^*2*^[*df* = 1] = 6.80, *p* = .009, *B* = 1.19, *O*.*R*. = 3.29, 95% *CI* [1.31, 8.26]). These results indicate that the PROGRESS program enhanced the size of student’s networks of developmental mentoring relationships from one to multiple mentors.

To further test the impact of PROGRESS on mentoring support, we conducted a series four of hierarchical logistic regression models predicting each of the four sources of mentoring support (i.e., faculty, graduate students, peers, & scientific professionals) from PROGRESS membership, controlling for college campus. Consistent with our expectations, the odds of having a faculty mentor were 3.5-times higher for PROGRESS members than control participants (*χ*^*2*^[*df* = 1] = 6.87, *p* = .009, *B* = 1.25, *O*.*R*. = 3.48, 95% *CI* [1.32, 9.19]). Analysis revealed a similar trend for having a peer mentor, but the trend did not exceed a conventional level of statistical significance (*χ*^*2*^[*df* = 1] = 2.99, *p* = .08, *B* = 0.75, *O*.*R*. = 2.12, 95% *CI* [0.89, 5.04]). PROGRESS and control members had equally low rates of being mentored by graduate students (*χ*^*2*^[*df* = 1] = 0.03, *p* = .88, *B* = 0.08, *O*.*R*. = 1.08, 95% *CI* [0.40, 2.97]) and scientific professionals outside of the university (*χ*^*2*^[*df* = 1] = 0.52, *p* = .47, *B* = 0.30, *O*.*R*. = 1.35, 95% *CI* [0.60, 3.01]).

Taken together, the complementary analyses indicate that PROGRESS members had, on average, larger networks of developmental mentoring relationships and were more likely to identify faculty and peers as sources of mentoring support.

#### Mentoring impacts on identity, interest, and persistence

Next, we assessed the degree to which mentoring support enhanced scientific identity, deep interest in earth and environmental sciences, and scientific persistence intentions through a series of three hierarchical regression models. The sources of mentoring support (i.e., faculty, graduate student, peer, and scientific professionals outside the university) were highly correlated with the size of the student’s network of developmental mentoring network (i.e., 0, 1, multiple). Therefore, we chose to proceed using only the sources of mentoring support as a predictive variable, as these provide a better insight into how specific kinds of mentoring support enhance student identity, motivation, and persistence intentions. In addition, we tested the indirect effects of PROGRESS membership and mentoring on interest and persistence through scientific identity.

In the first model, scientific identity was predicted from binary-coded indicators of the four sources of mentoring support (i.e., faculty, graduate students, peers, and scientific professionals outside the university) and PROGRESS membership, controlling for college campus (step-1). The analysis revealed that only faculty mentorship status predicted scientific identity, such that students with a faculty mentor had higher scientific identity scores compared to those without, see Table 2. In the second and third models, deep interest in earth and environmental sciences and persistence intentions were predicted from scientific identity (step-3), the four sources mentoring support and PROGRESS membership (step-2), and college campus (step-1). Faculty mentorship status positively predicted interest, while scientific identity positively predicted persistence intentions, see Table 2. Consistent with expectations, the analyses indicate that faculty mentorship is associated with higher levels of scientific identity and deep interest, while scientific identity was associated with persistence intentions. Taken together, these findings hint at the expected indirect effect of mentorship support on persistence intentions through scientific identity.

**Table 2:** Summary of the final step of hierarchical regression models predicting outcomes (science identity, deep interest, & persistence intentions) from relevant predictors and controls.

	Science Identity			Deep Interest			Persistence Intentions		
	*b*	*S*.*E*.	*β*	*b*	*S*.*E*.	*β*	*b*	*S*.*E*.	*β*
Intercept	4.77	.32		3.72	.88		3.59	.46	
College-2 vs. -1 (Binary-variable)	-0.21	.44	-.05	-0.24	.68	-.04	-0.11	.35	-.03
College-3 vs. -1 (Binary -variable)	-0.35	.32	-.13	0.58	.50	.14	0.10	.26	.04
College-4 vs. -1 (Binary -variable)	-0.38	.39	-.11	-0.29	.61	-.05	0.48	.32	.16
College-6 vs. -1 (Binary -variable)	0.16	.44	.04	-0.62	.68	-.10	0.40	.35	.11
College-6 vs. -1 (Binary -variable)	0.00	.46	.00	0.19	.72	.03	0.04	.37	.01
College-7 vs. -1 (Binary -variable)	-0.66	.45	-.17	-0.08	.71	-.01	0.20	.37	.06
PROGRESS status	0.18	.23	.07	-0.12	.36	-.03	-0.11	.19	-.05
Faculty mentor	0.89	.28	.35**	1.23	.45	.32**	-0.25	.24	-.11
Graduate Student mentor	-0.28	.31	-.09	0.13	.49	.03	0.02	.26	.01
Peer mentor	-0.07	.26	-.03	0.24	.41	.06	-0.04	.21	-.02
Scientific Professional mentor	0.14	.25	.05	0.10	.38	.03	-0.06	.20	-.03
Science Identity				0.04	.15	.02	0.50	.08	.56***

Notes: Correlation between interest and persistence intentions was *r* = .38; Model fit statistics: Science Identity Step-1 *F*(*6*, *109*) = 1.01, *p* = .43, *R*^*2*^ = .05, Step-2 *ΔF*(*5*, *104*) = 2.77, *p* = .02, *ΔR*^*2*^ = .11; Interest in Earth and Environmental Sciences Step-1 *F*(*6*, *109*) = 0.85, *p* = .53, *R*^*2*^ = .05, Step-2 *ΔF*(*5*, *104*) = 1.98, *p* = .09, *ΔR*^*2*^ = .08, Step-3 *ΔF*(*1*, *103*) = 0.05, *p* = .82, *ΔR*^*2*^ < .001; Persistence Intentions Step-1 *F*(6, 109) = 0.78, *p* = .59, *R*^*2*^ = .04, Step-2 *ΔF*(5, 109) = 0.15, *p* = .98, *ΔR*^*2*^ = .01, Step-3 *ΔF*(1, 103) = 39.34, *p* < .001, *ΔR*^*2*^ = .26; β = standardized coefficient, b = unstandardized coefficient, S.E. = standard error.

*p < .05

**p < .01

***p < .001

**Fig 2:**
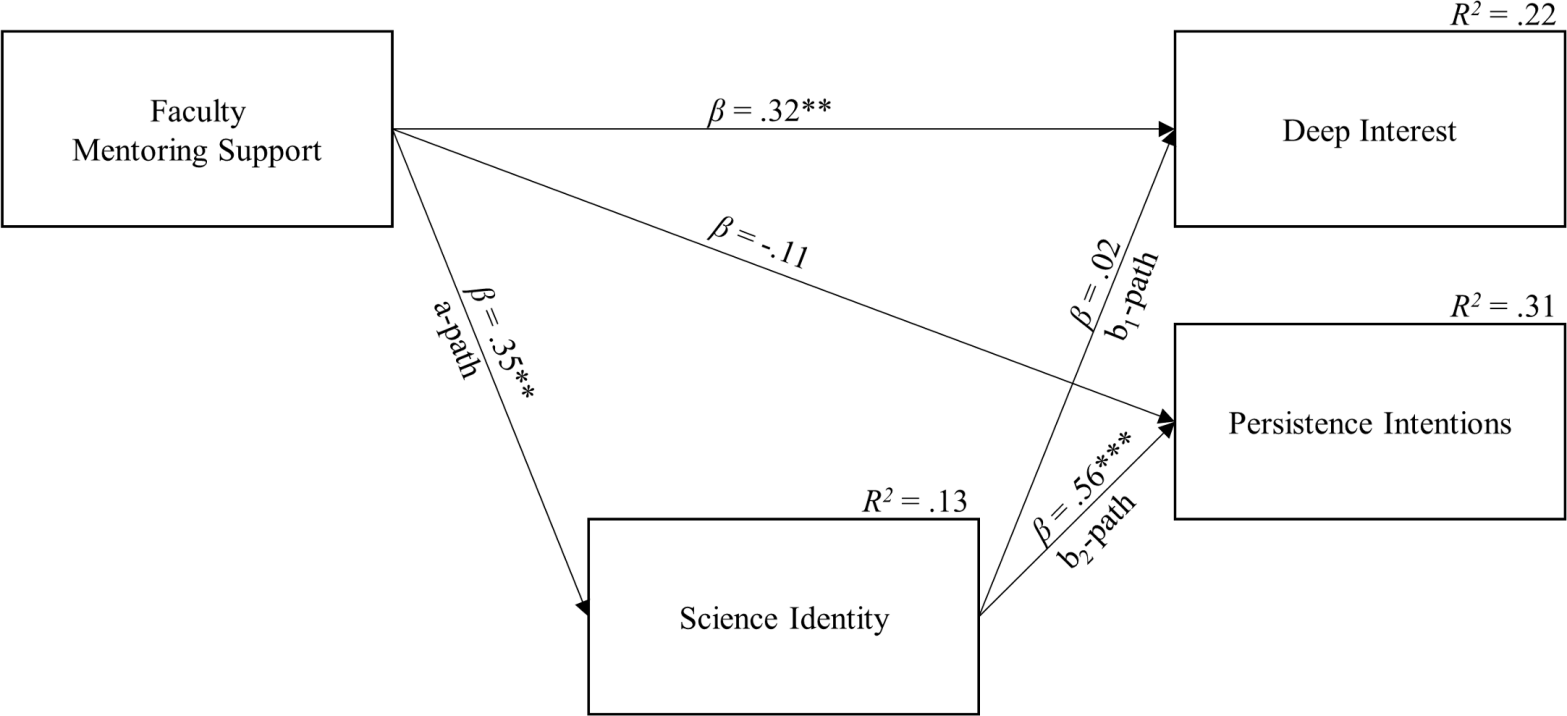
Estimated mediation howing the direct and indirect effects of mentoring support on motivation and persistence. Regression-based bootstrapped mediation models show a direct effect of faculty mentoring support (binary) on deep interest (outcome) and an indirect effect of faculty mentoring on persistence intentions (outcome) through science identity (mediator). *R*^*2*^ = proportion variance explained, *β* = standardized regression coefficient. *p < .05, **p < .01, ***p < .001.

We tested for mediation by estimating the bootstrapped (with 10,000 repetitions) bias corrected 95% confidence intervals around the indirect effect of faculty mentoring on persistence intentions through science identity using the PROCESS macro [74]. The analysis revealed a significant positive indirect effect of faculty mentorship on persistence intentions through scientific identity (*β*_*a×b Partial Standardized*_ = .41, *b*_*a×b*_ = 0.44, bias corrected 95% *CI* [0.18, 0.79]). Thus, participants with a faculty mentor had persistence intentions 0.41 standard deviations higher compared to those without due to the indirect effect of mentorship on scientific identity, see Fig 2.

Taken together, the analyses indicate that support from a faculty mentor is associated with higher levels of scientific identity development and deeper interest in earth and environmental sciences. Furthermore, the conditional process analysis indicated that faculty support is associated with higher scientific persistence intentions, through scientific identity development.

## Discussion

Female undergraduates can experience social barriers that undermine their scientific identity development, motivation, and persistence in STEM education and career pathways [3, 7]. The present study followed first- and second-year female undergraduate STEM majors in the context of the PROGRESS program, a mentoring program based on the success of the Earth Science Women’s Network (an organization that supports peer mentoring and early-career professional development for earth and environmental scientists; [57]). This study aimed to answer two critical research questions:1) to what degree did the PROGRESS program broaden the participating undergraduate’s network of developmental mentoring relationships?; and 2), what were the unique effects of mentoring support on identity development, motivation, and persistence intentions? Importantly, the use of a prospective propensity score matching design allowed us to compare the PROGRESS group to a matched treatment-as-usual control group and estimate a conditionally unbiased treatment effect (i.e., estimate an unbiased treatment effect, assuming the relevant confounding variables were used in matching) [60, 61]. The longitudinal design ensured that the intervention occurred prior to assessment of the outcomes. And we relied upon an empirically grounded persistence framework to test the conditional processes (i.e., mediated pathway) through which PROGRESS was expected to impact motivation and persistence [7, 51].

Consistent with our expectations, undergraduate women in PROGRESS were more likely to have multiple mentors and were more likely to receive support from faculty and peer mentors compared to their matched female controls. These results are consistent with research showing that, in the absence of a deliberative mentoring intervention, most first-year students (in this case 48%) tend to seek out a single mentor [34]. We were interested in the degree to which different sources of mentoring support enhance integration into the scientific community [7]. Consistent with our expectations, students receiving mentoring support from faculty members reported having higher levels of scientific identity and reported higher levels of interest in the earth and environmental sciences compared to students without faculty mentors. Partially consistent with our expectations, scientific identity mediated the effect of faculty mentoring support on scientific career persistence intentions.

Taken together, these findings extend the mentoring literature in several important ways. First, much of the research on mentoring undergraduates has focused on upper-division science majors in the context of multicomponent science training programs (e.g., Meyerhoff program or REUs; [9], which limits understanding the unique impact of mentoring. By contrast, the present study focused exclusively on an informal mentorship program for lower-division female science majors. Consistent with previous research, we found that mentoring support from faculty members benefits early-undergraduate women by strengthening their scientific identity, but did not observe benefits from graduate student and peers mentoring support. Prior research on peer or step-ahead mentoring (e.g., graduate student) in the sciences has primarily been in the context of research experiences [75]. Therefore, it is possible that aspects of the research experience context, such as the focus on transfer of disciplinary knowledge and skills, make peer and graduate student mentoring particularly beneficial to a protégé’s ability to gain from the research experience.

Previous research studies of single-component mentoring programs have primarily focused on struggling students at a single institution and often lack an adequate comparison group to create an experimental design (e.g., non-equivalent comparison groups or no comparison group) [11, 23–27]. By contrast, this study focused on average achieving and high achieving first- and second-year female science majors at multiple universities and used a prospective, PSM, longitudinal design to assess the unique benefits of mentoring. Consistent with mentoring and psychological theory, our data indicate that mentoring support has beneficial effects on self-beliefs (scientific identity), deep interest (i.e., motivation), and persistence intentions, which have been shown to lead to longer-term persistence and achievement outcomes [7, 43].

Although the present study addressed several gaps in the mentoring literature, there are at least two distinct limitations to our inferences. First, these data only follow changes in college women’s experiences across a total of six months out of a single academic year. It is probable that the developmental needs of aspiring scientists change as they advance toward graduation and more advanced career pursuits. Therefore, we expect that the observed benefits of receiving mentoring support from a faculty member as well as the lack of observed benefits of receiving mentoring support from other sources will change over time. The focus on early tenure students and the relatively short timeframe limit our ability to examine dynamic changes in mentoring benefits in the different phases of undergraduate tenure and over time in this report. However, because the PROGRESS program is ongoing, future studies will examine the changing benefits of faculty and near-peer mentors for female science majors over a longer period of time.

A second potential limitation concerns the design of the control group. The current study used a PSM approach to construct an “inactive” or treatment as usual control. Comparisons with an inactive control group allow for an assessment of the overall benefits of a treatment package, but do not allow inferences about individual components of a treatment package [55, 56]. In addition, the PSM methodology provides unbiased estimates of treatment effects when controlling for observed confounding variables [60]. Statistical bias would undermine the present findings if confounding variables were omitted from our PSM analysis. However, we designed our matching survey to include a large number of known or theoretically suspected confounding variables. Therefore, we do not believe that many relevant variables that could pose bias are playing a role in the observed effects.

## Conclusions

Mentoring and psychological theories suggest that women in some scientific disciplines face unique challenges to persistence and benefit from having larger networks of developmental mentoring relationships. We examined the benefits a novel informal mentoring program (PROGRESS) aimed at supporting first- and second-year female STEM majors’ pursuit of scientific career pathways, with particular focus on careers in earth an environmental sciences.

1. We found that PROGRESS expanded participants’ networks of developmental mentoring relationships compared to a matched control group. In particular, PROGRESS members were more likely to receive mentoring support from faculty members and peers compared to matched controls.
2. Mentoring support from a faculty member strengthened participant’s scientific identity, motivation, and (indirectly) persistence intentions. These factors have been shown by prior studies to lead to longer-term persistence and achievement outcomes.

## Supporting information

S1 Table Summary of mentor support descriptive statistics as a function of PROGRESS status (*N* = 116). (PDF)

S2 Table Summary of differences between PROGRESS and matched group before and after propensity score matching. (PDF)

S3 Table Summary of correlations among PROGRESS status, mentoring support, science identity, deep interest, and persistence intentions (*N* = 116). (PDF)
