# Supplementary material for: Promoting professional identity, motivation, and persistence: Benefits of an informal mentoring program for female undergraduate students
Source: PLoS One. 2017 Nov 1;12(11):e0187531. doi: 10.1371/journal.pone.0187531 (PMC5665547; doi:10.1371/journal.pone.0187531)
Supplement: S1 Table — (PDF) [file pone.0187531.s001.pdf]

Promoting professional identity, motivation, and persistence: Benefits of an informal mentoring program for female undergraduate students

Participants

Sixty-nine percent of the PROGRESS (experimental) group had a closely matched counterpart in the control group (see propensity score matching section below for complete details). The sample was predominantly composed of U.S. nationals of European descent, and most spoke English as a first language, see Table 1.

Supporting Table 1. Summary of Descriptive Statistics as a Function of PROGRESS Status (*N* = 116).

Variables	Matched Control<br>*%*	PROGRESS<br>*%*
Foreign National status	7	7
European decent	74	79
African American	3	3
Asian	5	3
Latina	5	5
Native American/Pacific Islander/First Nation	9	14
Other	2	3
English as a first language	86	90
First year of college	43	50
Second year of college	57	50
Major=Agricultural Science	5	3

Variables	Matched Control<br>%	PROGRESS<br>%
Major=Biological / Life Sciences	35	35
Major=Engineering	26	22
Major=Mathematics Or Computer Science	10	7
Major=Natural / Geological Sciences	24	33

Notes: SAT / ACT equivalent scores for PROGRESS and control groups were (*M* = 1305.90 [86-91th percentile], *SD* = 127.83 and *M* = 1309.12 [86-91th percentile], *SD* = 117.74, respectively). Baseline Grade Point Average (GPA) scores for PROGRESS and control groups were (*M* = 3.67 [0-4 point scale], *SD* = 0.33 and *M* = 3.69 [0-4 point scale], *SD* = 0.41, respectively). *N* = total sample size, *M* = mean, *SD* = standard deviation.
