# Supplementary material for: Promoting professional identity, motivation, and persistence: Benefits of an informal mentoring program for female undergraduate students
Source: PLoS One. 2017 Nov 1;12(11):e0187531. doi: 10.1371/journal.pone.0187531 (PMC5665547; doi:10.1371/journal.pone.0187531)
Supplement: S2 Table — (PDF) [file pone.0187531.s002.pdf]

Promoting professional identity, motivation, and persistence: Benefits of an informal mentoring program for female undergraduate students

Propensity score matching (PSM)

PSM analysis was implemented in MatchIT software [1-3]. The 57 matching covariates included demographic characteristics (e.g., race/ethnicity), family characteristics (e.g., parental highest level of education), academic characteristics (e.g., GPA), psychological factors relating to interest and success in science (e.g., science self-efficacy), interest in earth systems and environmental sciences, interest in having a mentor, and interest in participating in the longitudinal study. Prior to matching, we evaluated missing data in the matching variables. We found that 18 of the 57 matching variables had an extremely small percentage of missing responses (average percept missing = 1.7%), however, Little's MCAR test [4] revealed that the data were missing completely at random, $\chi^2(df=555) = 527.21$, $p = .80$. Therefore, consistent with recommendations in PSM we imputed the mean for the missing values on the matching variable prior to PSM analysis [5]. We specified a PSM model using a 1:1 nearest neighbor approach without replacement, with a caliper set to 0.20 standard deviations of the logit of the estimated propensity score, and matched samples were restricted to participants inside the area of common support for both groups [5]. Finally, we only used matched pairs in which both the PROGRESS and matched control participant provided data for the outcome analysis, resulting in PROGRESS and control groups of equal size ($n_{PROGRESS} = 58$, $n_{Control} = 58$).

The quality of the match was evaluated based on plots of the propensity score distribution in each group, an examination of the reduction in bias for each covariate, and reduction in bias

for the overall model. After matching, all of the individual covariates used in matching showed acceptable balance across PROGRESS and control groups (i.e., Cohen's *d* values < 0.25).

Supporting Table 2. Summary of differences between PROGRESS and matched group before and after propensity score matching.

	$M_{PROGRESS}$		$M_{Control}$		$SD_{Control}$		*Cohen's d Differeence*	
Source	*Before*	*After*	*Before*	*After*	*Before*	*After*	*Before*	*After*
Propensity	0.50	0.39	0.19	0.38	0.18	0.20	1.19	0.05
Heterosexual status	0.16	0.14	0.15	0.15	0.36	0.36	0.04	-0.05
Parental Status	0.04	0.03	0.07	0.00	0.25	0.00	-0.16	0.18
Do you plan to have children (or more children) in the future?	2.38	2.25	2.16	2.24	1.17	1.13	0.16	0.01
U.S. or Foreign national status	0.06	0.07	0.05	0.07	0.22	0.25	0.04	0.00
U.S. national (European decent)	0.76	0.80	0.79	0.75	0.41	0.44	-0.07	0.12
U.S. national (African)	0.04	0.03	0.04	0.03	0.20	0.18	-0.03	0.00
U.S. national (Asian)	0.05	0.03	0.07	0.05	0.25	0.22	-0.08	-0.08
U.S. national (Latina)	0.04	0.05	0.07	0.05	0.25	0.22	-0.16	0.00
U.S. national (Native American/Pacific Islander/First Nation)	0.11	0.14	0.10	0.08	0.30	0.28	0.05	0.16
U.S. national (Other)	0.05	0.03	0.03	0.02	0.17	0.13	0.10	0.08
Is English your first language?	0.90	0.90	0.91	0.86	0.28	0.35	-0.04	0.11
Do you identify as having a physical disability?	0.03	0.00	0.01	0.00	0.10	0.00	0.10	0.00
Are you a veteran or currently in the military?	0.01	0.02	0.02	0.00	0.15	0.00	-0.10	0.15
Parental highest level of education	6.43	6.53	6.21	6.44	1.34	1.16	0.16	0.06
Has anyone in your family completed a degree in the geosciences?	0.14	0.17	0.16	0.20	0.37	0.41	-0.06	-0.10
Total annual income of the family that raised you.	4.45	4.66	4.63	4.47	1.33	1.26	-0.12	0.13
Financial resources for college: School scholarship	0.65	0.63	0.61	0.66	0.49	0.48	0.08	-0.07

Source	$M_{PROGRESS}$ *Before*	$M_{PROGRESS}$ *After*	$M_{Control}$ *Before*	$M_{Control}$ *After*	$SD_{Control}$ *Before*	$SD_{Control}$ *After*	*Cohen's d Differeence Before*	*Cohen's d Differeence After*
What kind of financial resources support your college studies? Job off campus	0.11	0.10	0.18	0.08	0.38	0.28	-0.21	0.05
What kind of financial resources support your college studies? External scholarship	0.24	0.27	0.31	0.31	0.46	0.46	-0.16	-0.08
What kind of financial resources support your college studies? Parental or other family support	0.65	0.66	0.69	0.66	0.46	0.48	-0.09	0.00
What kind of financial resources support your college studies? Work-study	0.11	0.10	0.12	0.14	0.32	0.35	-0.01	-0.11
What kind of financial resources support your college studies? Residence assistantship	0.00	0.00	0.02	0.00	0.15	0.00	-	-
What kind of financial resources support your college studies? Partner's employment	0.00	0.00	0.00	0.02	0.07	0.13	-	-
What kind of financial resources support your college studies? Loans	0.45	0.39	0.41	0.42	0.49	0.50	0.08	-0.07
What kind of financial resources support your college studies? Other job on campus	0.15	0.07	0.06	0.10	0.23	0.30	0.26	-0.09
What kind of financial resources support your college studies? Savings	0.28	0.27	0.29	0.31	0.46	0.46	-0.04	-0.08
What kind of financial resources support your college studies? Other	0.16	0.14	0.05	0.10	0.22	0.30	0.30	0.09
Full-time student status	1.00	1.00	1.00	1.00	0.00	0.00	-	-
College-1	0.18	0.20	0.25	0.24	0.43	0.43	-0.20	-0.09
College-2	0.10	0.08	0.09	0.12	0.28	0.33	0.04	-0.11
College-3	0.24	0.29	0.23	0.24	0.42	0.43	0.02	0.12
College-4	0.15	0.14	0.14	0.14	0.35	0.35	0.02	0.00
College-5	0.13	0.12	0.11	0.08	0.31	0.28	0.06	0.10

	$M_{PROGRESS}$		$M_{Control}$		$SD_{Control}$		Cohen's d Differeence	
Source	Before	After	Before	After	Before	After	Before	After
College-6	0.10	0.05	0.12	0.10	0.33	0.30	-0.07	-0.17
College-7	0.11	0.12	0.06	0.08	0.24	0.28	0.16	0.11
First year of college	0.46	0.49	0.55	0.42	0.50	0.50	-0.18	0.14
Second year of college	0.54	0.51	0.45	0.58	0.50	0.50	0.18	-0.14
Are you attending college in-state or out of state?	0.45	0.42	0.37	0.36	0.48	0.48	0.17	0.14
Major=Agricultural Science	0.05	0.03	0.02	0.05	0.15	0.22	0.12	-0.08
Major =Biological / Life Sciences (e.g. Biology, Pre Medicine, Veterinary, etc..)	0.34	0.36	0.35	0.34	0.48	0.48	-0.03	0.04
Major =Engineering	0.19	0.22	0.28	0.25	0.45	0.44	-0.24	-0.09
Major =Mathematics Or Computer Science	0.06	0.07	0.09	0.12	0.29	0.33	-0.12	-0.21
Major =Natural / Geological Sciences (e.g., Chemistry, Atmospheric Sciences, etc..)	0.36	0.32	0.25	0.24	0.43	0.43	0.23	0.18
Have you decided on a major (or double major)?	0.84	0.80	0.81	0.80	0.39	0.41	0.07	0.00
Most recent GPA for matching	3.69	3.67	3.64	3.69	0.39	0.40	0.14	-0.04
SAT combined / ACT Equiv. for matching	1311.49	1306.64	1288.09	1307.61	125.73	117.30	0.19	-0.01
Did you take an AP or IB course in science?	0.76	0.78	0.75	0.75	0.43	0.44	0.02	0.08
Home=Urban	0.18	0.22	0.19	0.19	0.39	0.39	-0.03	0.09
Home=Rural	0.19	0.19	0.22	0.20	0.42	0.41	-0.09	-0.04
Home=Suburban	0.64	0.59	0.59	0.61	0.49	0.49	0.10	-0.04
Participated in a summer science program	0.35	0.32	0.25	0.36	0.44	0.48	0.20	-0.07
Been a member of an honor society	0.71	0.69	0.69	0.69	0.46	0.46	0.04	0.00
Participated in a science competition, a science fair, or professional research conference	0.38	0.34	0.38	0.31	0.49	0.46	0.00	0.07
Interest in having a mentor on your campus?	0.96	0.95	0.91	0.97	0.28	0.18	0.26	-0.09

Source	$M_{PROGRESS}$ Before	$M_{PROGRESS}$ After	$M_{Control}$ Before	$M_{Control}$ After	$SD_{Control}$ Before	$SD_{Control}$ After	Cohen's d Differeence Before	Cohen's d Differeence After
Interested in participating in long-term study?	4.11	4.03	3.84	4.05	0.92	0.75	0.30	-0.02
Science is fun[a]	4.59	4.56	4.48	4.61	0.61	0.53	0.21	-0.10
I am good at science[b]	4.25	4.19	4.07	4.22	0.64	0.59	0.32	-0.06
It would be thrilling to make a scientific discovery[c]	4.79	4.75	4.75	4.76	0.48	0.47	0.11	-0.01
I enjoy math[d]	3.63	3.66	3.79	3.75	1.01	1.11	-0.16	-0.08
Mathematics is one of my best subjects[e]	3.31	3.36	3.50	3.39	1.19	1.31	-0.15	-0.03
Being a woman is an important part of my identity[f]	4.24	4.25	4.26	4.42	0.83	0.75	-0.03	-0.23
I like being a woman[f]	4.24	4.32	4.35	4.34	0.73	0.71	-0.14	-0.03
My ethnicity is an important part of my identity[g]	3.05	3.20	3.26	3.31	1.02	1.05	-0.19	-0.09
I can see how majoring in the geosciences relates to my future education and occupational goals[h]	4.02	3.96	3.75	4.03	1.02	0.91	0.27	-0.07
How much do you know about the geosciences? (e.g., have taken courses)[i]	2.93	2.88	2.65	2.75	1.21	1.17	0.25	0.12
How much do you know about OCCUPATIONS in the geosciences?[i]	2.50	2.42	2.30	2.24	1.09	0.99	0.17	0.16
People who are good at science are [feminine -- masculine][j]	2.81	2.85	2.88	2.92	0.45	0.53	-0.16	-0.17
Science is a domain that is [feminine -- masculine][j]	2.50	2.56	2.59	2.51	0.59	0.68	-0.14	0.08
When I think of people who are very good at science, I think of [feminine -- masculine][j]	2.53	2.61	2.60	2.54	0.68	0.73	-0.09	0.08
The field of geoscience is [feminine -- masculine][j]	2.55	2.58	2.66	2.63	0.60	0.67	-0.18	-0.08
Everyone (male or female) has a fair shot at educational opportunities[k]	2.94	3.10	3.13	3.02	1.28	1.36	-0.17	0.08

	$M_{PROGRESS}$		$M_{Control}$		$SD_{Control}$		*Cohen's d Differeence*	
Source	*Before*	*After*	*Before*	*After*	*Before*	*After*	*Before*	*After*
Having a social impact is important to my choice of major[l]	4.19	4.19	4.11	4.24	0.82	0.77	0.10	-0.06
Mastering tasks is what I value in my education[m]	4.11	4.14	4.20	4.10	0.74	0.82	-0.12	0.05
I am drawn to majors that require hands-on, physical work[n]	4.11	4.02	4.05	4.12	0.88	0.81	0.08	-0.12
I am an outdoorsy person[n]	4.02	4.00	3.90	3.81	1.10	1.15	0.11	0.17
I am confident that I can use technical science skills (use of tools, instruments, and/or techniques)[o]	4.14	4.03	4.07	4.02	0.75	0.86	0.09	0.02
I consider myself to be a people person[p]	3.80	3.76	3.78	3.61	1.04	1.13	0.02	0.16
Discrimination against women is no longer a problem in the United States[q]	1.76	1.85	1.90	1.73	0.85	0.76	-0.18	0.16
Women are just as capable of thinking logically as men[q]	4.88	4.86	4.83	4.81	0.54	0.60	0.16	0.15
How interested are you in taking courses in the geosciences?[r]	3.73	3.68	3.47	3.68	1.22	1.20	0.22	0.00
How interested are you in pursuing a GEOSCIENCE DEGREE? [r]	3.14	3.08	2.90	2.85	1.42	1.44	0.18	0.18
How interested are you in pursuing a GEOSCIENCE GRADUATE DEGREE?[r]	2.80	2.71	2.51	2.49	1.32	1.34	0.21	0.16
How likely are you to pursue a GEOSCIENCE OCCUPATION?[r]	2.96	2.93	2.81	2.75	1.36	1.27	0.12	0.14
If you do poorly on a test people will assume that it is because you are a woman[s]	2.10	2.14	2.01	1.98	1.03	1.01	0.08	0.14

Notes: $M_{PROGRESS}$ *Before/After* = mean value in the PROGRESS group before/after matching; $M_{Control}$ *Before/After* = mean value in the PSM matched control group before/after matching; $SD_{Control}$ *Before/After* = standard deviation value in the PSM matched control group before/after matching; *Cohen's d difference* Before/After = standardized mean difference between PROGRESS and control

group before/after matching. It is standard practice to flag Cohen’s d difference values $\geq$ .20 as indicating imbalance across groups, but none of the values exceeded this limit after matching.

[a]Indicator of positive attitude toward science (adapted from original source) [6].

[b]Indicator of science self-concept [7].

[c]Indicator of science community values [8].

[d]Indicator of positive attitude toward mathematics [6].

[e]Indicator of mathematics self-efficacy [9]

[f]Indicator of gender identity [10].

[g]Indicator of ethnic identity [10].

[h]Indicator of science utility values [11]

[i]Indicator of geoscience career path knowledge.

[j]Indicator of stereotypes about geoscience [12].

[k]Indicator of gender-specific system justification [13].

[l]Indicator of communal values [14].

[m]Indicator of mastery goal orientation [15].

[n]Indicator of outdoors behavioral interest.

[o]Indicator of science self-efficacy [16].

[p]Indicator of communal goal attainment values [14].

[q]Indicator of perceptions of sexism [17].

[r]Indicator of deep interest in geosciences [11].

[s]Indicator of gender-based stereotype threat [18].

References

1. Ho DE, Imai K, King G, Stuart EA. Matching as nonparametric preprocessing for reducing model dependence in parametric causal inference. Political Analysis. 2007;15(3):199-236.
2. Ho DE, Imai K, King G, Stuart EA. MatchIt: Nonparametric preprocessing for parametric causal inference. J Stat Softw. 2011;42(8).
3. Thoemmes F. An SPSS R menu for propensity score matching. 2012.
4. Little RJA. A test of missing completely at random for multivariate data with missing values. Journal of the American Statistical Association. 1988;83(404):1198-202.
5. Pan W, Bai H. Propensity score analysis: Fundamentals and developments. New York, NY: Guilford Press; 2015.
6. Scott A, Martin A. Gender and racial stereotype endorsement and implications for STEM outcomes among high-achieving underrepresented adolescent females. 2013.
7. Marsh HW, Abduljabbar AS, Abu-Hilal MM, Morin AJ, Abdelfattah F, Leung KC, et al. Factorial, convergent, and discriminant validity of timss math and science motivation measures: A comparison of Arab and Anglo-Saxon countries. Journal of Educational Psychology. 2013;105(1):108.
8. Estrada M, Woodcock A, Hernandez PR, Schultz PW. Toward a model of social influence that explains minority student integration into the scientific community. Journal of Educational Psychology. 2011;103(1):206-22.
9. Walton GM, Cohen GL, Cwir D, Spencer SJ. Mere Belonging: The Power of Social Connections. Journal of Personality and Social Psychology. 2011;102(3):513-32.
10. Schmader T. Gender identification moderates stereotype threat effects on women's math performance. Journal of Experimental Social Psychology. 2002;38(2):194-201.
11. Hulleman CS, Godes O, Hendricks BL, Harackiewicz JM. Enhancing interest and performance with a utility value intervention. Journal of Educational Psychology. 2010;102(4):880-95.
12. Stout JG, Dasgupta N, Hunsinger M, McManus MA. STEMing the tide: Using ingroup experts to inoculate women's self-doncept in science, technology, engineering, and mathematics (STEM). Journal of Personality and Social Psychology. 2011;100(2):255-70.
13. Jost JT, Kay AC. Exposure to benevolent sexism and complementary gender stereotypes: consequences for specific and diffuse forms of system justification. Journal of personality and social psychology. 2005;88(3):498.
14. Diekman AB, Clark EK, Johnston AM, Brown ER, Steinberg M. Malleability in Communal Goals and Beliefs Influences Attraction to STEM Careers: Evidence for a Goal Congruity Perspective. Journal of Personality and Social Psychology. 2011;101(5):902-18.
15. Elliot AJ, Church MA. A hierarchical model of approach and avoidance achievement motivation. Journal of Personality and Social Psychology. 1997;72(1):218-32.
16. Chemers MM, Zurbriggen EL, Syed M, Goza BK, Bearman S. The role of efficacy and identity in science career commitment among underrepresented minority students. Journal of Social Issues. 2011;67(3):469-91.

17. Swim JK, Aikin KJ, Hall WS, Hunter BA. Sexism and Racism - Old-Fashioned and Modern Prejudices. Journal of Personality and Social Psychology. 1995;68(2):199-214.
18. Woodcock A, Hernandez PR, Estrada M, Schultz PW. The consequences of chronic stereotype threat: Domain disidentification and abandonment. Journal of Personality and Social Psychology. 2012;103(4):635-46.
