# Supplementary material for: Promoting professional identity, motivation, and persistence: Benefits of an informal mentoring program for female undergraduate students
Source: PLoS One. 2017 Nov 1;12(11):e0187531. doi: 10.1371/journal.pone.0187531 (PMC5665547; doi:10.1371/journal.pone.0187531)
Supplement: S3 Table — (PDF) [file pone.0187531.s003.pdf]

Promoting professional identity, motivation, and persistence: Benefits of an informal mentoring program for female undergraduate students

Supporting Table 3. Summary of correlations among PROGRESS status, mentoring support, science identity, deep interest, and persistence intentions (*N* = 116)

	Variable	*M*	*SD*	*1.*	*2.*	*3.*	*4.*	*5.*	*6.*	*7.*	*8.*	*9.*	*10.*
1.	PROGRESS status	0.50	0.50	--									
2.	Mentor network: 1 mentor	0.88	0.33	.05	--								
3.	Mentor network: multiple mentors[a]	0.56	0.50	.24*	--	--							
4.	Faculty mentor status	0.36	0.48	.25**	.28**	.54***	--						
5.	Graduate student mentor status	0.16	0.37	.02	.16	.17	.01	--					
6.	Peer mentor status	0.71	0.46	.15	.58***	.31**	.21*	-.18	--				
7.	Scientific professional mentor status	0.32	0.47	.06	.25**	.47***	-.02	.20*	.08	--			
8.	Science identity	4.91	1.21	.15	.10	.16	.34***	-.06	.08	.03	--		
9.	Deep interest in earth and environmental sciences	4.53	1.84	.06	-.02	.32***	.28**	.03	.13	.03	.10	--	
10.	Persistence intentions	6.00	1.08	.01	-.08	.06	.09	-.03	-.03	-.01	.52***	.21*	--

Notes: [a]Indicator variable for Mentor network: multiple mentors had $n = 102$ since the reference group was students with 1-mentor (i.e., students with no mentors were excluded). All indicator variables were dummy-coded.

*p<.05, **p<.01, ***p<.001
